# Impact of Nutrition-Based Interventions on Athletic Performance during Menstrual Cycle Phases: A Review

**DOI:** 10.3390/ijerph18126294

**Published:** 2021-06-10

**Authors:** Macy M. Helm, Graham R. McGinnis, Arpita Basu

**Affiliations:** Department of Kinesiology and Nutrition Sciences, University of Nevada, Las Vegas, NV 89154, USA; helmm1@unlv.nevada.edu (M.M.H.); graham.mcginnis@unlv.edu (G.R.M.)

**Keywords:** menstrual cycle, female athlete, exercise performance, rehydration, electrolytes, vitamins, minerals, phytochemicals

## Abstract

Despite the steady increase in female participation in sport over the last two decades, comprehensive research on interventions attenuating the influence of female menstrual physiology on performance remains scarce. Studies involving eumenorrheic women often only test in one menstrual phase to limit sex hormone variance, which may restrict the application of these findings to the rest of the menstrual cycle. The impacts of nutrition-based interventions on athletic performance throughout the menstrual cycle have not been fully elucidated. We addressed this gap by conducting a focused critical review of clinical studies that reported athletic outcomes as well as menstrual status for healthy eumenorrheic female participants. In total, 1443 articles were identified, and 23 articles were included. These articles were published between 2011 and 2021, and were retrieved from Google Scholar, Medline, and PubMed. Our literature search revealed that hydration-, micronutrient-, and phytochemical-based interventions can improve athletic performance (measured by aerobic capacity, anaerobic power, and strength performance) or attenuate exercise-induced damage (measured by dehydration biomarkers, muscle soreness, and bone resorption biomarkers). Most performance trials, however, only assessed these interventions in one menstrual phase, limiting the application throughout the entire menstrual cycle. Improvements in athletic performance through nutrition-based interventions may be contingent upon female sex hormone variation in eumenorrheic women.

## 1. Introduction

Female participation in sport, as a mode of recreation and profession, has been increasing in the United States and worldwide throughout the last two decades. After London won the 2012 Olympic Games bid in 2005, England saw an increase of one million women participating in sport and physical activity [1]. From 2011 to 2012, female participation in soccer increased by 43% in Australia [1]. In the United States, more than three million girls participate in high school athletics, and 46% of athletic collegiate scholarships are awarded to female recipients [1]. The 2016 Olympic Games had a historic 5059 female athletes, equaling 45% of the participants [2].

Despite this increasing exercise involvement, research supporting the optimization of female athletes and their performance is comparatively sparse. The menstrual cycle has been reported to influence athletic performance [3,4] and exercise metabolism [5]. Menstrual hormones also impact hydration mechanisms [6], which may have implications for female athletes and recreational exercisers. For eumenorrheic women, the menstrual cycle is an average of 28 days, characterized as two stages—follicular and luteal. The follicular phase begins on the first day of menses and ends at ovulation. The luteal phase begins after ovulation and ends on the first day of menses. These two stages can be characterized by reproductive hormone fluctuations [7]. Throughout the rise and fall of reproductive hormone concentrations within the menstrual cycle, the early follicular stage has a lower concentration of estrogen, which increases halfway through the phase and peaks prior to ovulation [7]. Estrogen and progesterone are both elevated in the luteal stage [7,8]. In a 28-day cycle, the early follicular phase is days 1–9, the late follicular phase is days 10–14, the early luteal phase is days 15–17, the mid-luteal phase is days 18–23, and the late luteal phase is days 24–28. Due to these hormone fluctuations, women are often tested in exercise science research during the early follicular phase to reduce hormone variance [8].

The historic lack of inclusion of women in sport and exercise research trials because of hormonal variability makes it difficult to accurately determine the implications of the cyclical menstrual cycle on performance [8]. Studies that do include women often test during the follicular phase of menstruation [8], when endocrine hormones are the least variable. Although testing exclusively in the follicular phase may reduce variability among research outcomes, the application of these findings for eumenorrheic athletes and recreational exercisers is inherently limited. By only having performance recommendations based upon one phase of the menstrual cycle, women are consequently left blinded to the optimization of training or performing during the rest of their menstrual cycle. Nearly 40% of surveyed women who engage regularly in exercise reported a belief that their menstrual cycle negatively impacted exercise training and performance [8], thus creating the necessity to expand exercise performance investigations on mechanisms impacted by intra-cycle fluctuations. 

Menstrual hormones are speculated to impact thermoregulation [9], fluid balance [6], blood plasma volume [10], and water and electrolyte regulation [11]. Blood plasma volume and fluid balance have a direct relationship with exercise performance. A decrease in body mass of 2% due to dehydration can impede athletic performance [12], and hypohydration at the start of exercise has been shown to reduce sport-specific performance [13]. This is further exacerbated by the fact that sweat rate is modulated by the menstrual cycle, and is increased during the luteal compared to the follicular phase [14]. Given the relationship between menstruation and fluid balance, rehydration-based strategies should consider the menstrual phase during which a female athlete or recreational exerciser is rehydrating. 

Analogous to hydration levels and rehydration therapies, iron status and subsequent supplementation impact athletic performance. Iron status varies throughout the menstrual cycle, and biochemical markers (i.e., serum iron and transferrin saturation) are significantly associated with the menstrual cycle: the values are lowest during menses [15]. In a representative sample of eumenorrheic women, the diagnosis of iron deficiency was more likely during menses than the luteal phase, indicating a significant reduction in serum ferritin or mean corpuscular volume [15]. Even without clinical iron deficiency, iron supplementation has been shown to improve distance running performance by reducing perceived fatigue and mood disturbances [16]. Iron stores also play a role in energy levels, and studies have shown that iron supplementation for non-anemic women can reduce unexplained fatigue [17], which could improve performance because mental fatigue is associated with poor physical performance [18]. Iron metabolism plays a role in both endurance performance and recovery from resistance training [19]; however, studies linking the female endocrine hormone fluctuation to performance outcomes concerning iron stores are nascent.

Athletic performance additionally relies on adequate calcium stores for the maintenance of bone health, which has become increasingly associated with energy availability in female athletes. A reduction of 15 kcal per kilogram of lean body mass has been shown to decrease bone formation [20], increasing the risk of stress fracture injuries. A better understanding of calcium supplementation throughout the menstrual cycle could offer an enhancement of training programs to maximize bone metabolism while minimizing the risk of stress fracture injury due to fluctuations in female endocrine hormones.

Various studies have also reported the influence of phytochemical-based dietary supplements on athletic performance. Bioactive compounds, such as those in tart cherry juice [21], aronia-citrus juice [22], mixed flavonoids [23], phytoestrogens [24], and mango leaf extract [25], have been shown to modulate exercise-induced muscle damage and oxidative stress in aerobic exercise trials with men and women. Other interventions, such as with purple grape juice [26] and pomegranate extract [27], also have positive effects on female exercise performance, as measured by increased running time to exhaustion. These aforementioned studies, however, did not report the menstrual phase for the participants. Although interest in the supplementation of polyphenols is growing, substantial data on these compounds influencing performance outcomes related to the cycle of female endocrine hormones remain scarce.

The cyclical nature of the hormonal variation throughout the menstrual cycle poses a challenge for optimal performance and thus creates a unique opportunity for female athletes and recreational exercisers to utilize nutritional interventions to improve athletic performance. Thus, we conducted the present review to synthesize and describe the most recent literature on the impact of nutrition-based interventions on female athletic performance during specific menstrual phases.

## 2. Materials and Methods

**Figure 1:**
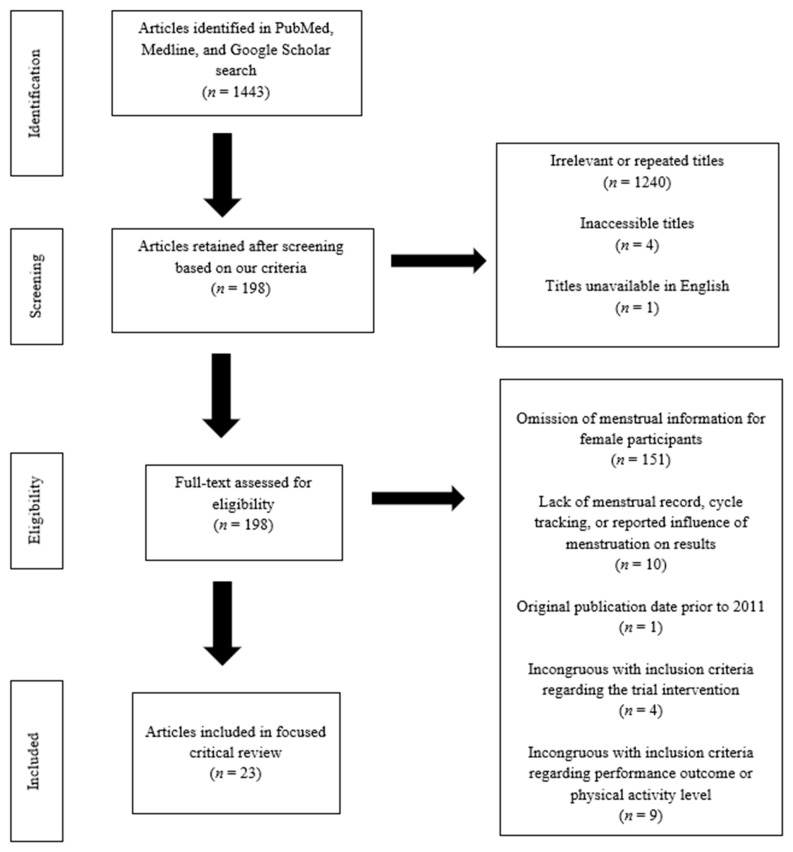
Flow diagram illustrating the search and selection of published articles using our inclusion and exclusion criteria.

A literature search was conducted using the databases PubMed, Medline, and Google Scholar. The search focused on clinical trials examining the effects of rehydration therapies, vitamin and micronutrient interventions, and dietary bioactive compound supplementation on exercise performance outcomes for physically active eumenorrheic female subjects. Selected articles were those published from 2011 to 2021. Search terms included “female athlete”, “exercise performance”, “menstrual cycle”, “follicular phase”, “fluid balance”, “dehydration”, “hydration”, “electrolyte”, “iron”, “calcium”, “micronutrients”, “vitamins”, “minerals”, “dietary bioactive compounds”, “polyphenols”, “flavonoids”, “omega fatty acids”, “nutraceuticals”, “herbs”, and “botanicals”. This search yielded a total of 1443 articles (Figure 1), which were first assessed by their title and abstract, and secondly by the subjects’ sex and age, as per our predetermined eligibility criteria. Articles whose title and subjects’ sex and age matched these criteria were then reviewed by examining their full texts. Ultimately, 23 articles were eligible and included. All authors agreed on the inclusion of the articles. 

Inclusion criteria included: (1) female subjects; (2) reproductive age; (3) the recording of menstrual phase or cycle; (4) physically active participants; (5) carbohydrate and/or electrolyte rehydration, micronutrient, or phytochemical-based dietary supplement intervention; (6) measurement of athletic performance-related outcomes—either aerobic or anaerobic cardiovascular fitness, muscular strength or endurance, or attenuation of exercise-induced physiological stress. Exclusion criteria included: (1) non-human models; (2) age outside of reproduction; (3) participants with chronic conditions; (4) omission of participant menstrual information; (5) non-active participants; (6) measurement of non-athletic performance-related outcomes; and (7) non-rehydration, non-micronutrient, and non-phytochemical-based interventions (e.g., creatine, whey protein, amino acid supplementation). M.M.H. executed the search and collated the articles for data collection and review. A.B. assisted with the search and confirmed that the selected articles met the eligibility criteria.

To determine the impact of the nutrition-based interventions on athletic performance outcomes, we determined a net increase or decrease in athletic ability using the data reported in the results sections of the included articles. Various athletic performance outcomes have been reported; therefore, the net increase or decrease in athletic ability is unique to each study. 

## 3. Results

Of the 23 manuscripts included, 22 were randomized trials, with 19 studies having at least one control or placebo group.

### 3.1. Hydration Interventions 

As summarized in Table 1, eleven studies assessing athletic outcomes related to hydration-based interventions originated from the United Kingdom [28], the United States [29,30], Greece [31], Japan [32], Hong Kong [33], China [34], Mexico [35], Canada [36], Australia [37], and New Zealand [38]. Four studies included recreationally active women [28,30,34,36]; two studies included moderately trained runners [31,33]; two studies included aerobically conditioned participants [29,37]; one study included amateur spinners [35]; one study included soccer players [38]; and one study included participants not trained in endurance activities [32]. Nine studies measured performance outcomes in the follicular phase [29,30,31,32,33,34,35,36,37] related to physiologic markers of dehydration or exercise capacity and tolerance. One study measured sprint performance, the Loughborough Soccer Passing Test, and physiologic markers of dehydration in the luteal phase [38]. The eleventh study measured fluid and electrolyte balance in two phases of the menstrual cycle [28]. 

The one study reporting outcomes for two menstrual phases utilized a commercial carbohydrate and electrolyte beverage (6.4% carbohydrate, 25 millimolar sodium, and 3.5 millimolar potassium) to rehydrate participants in their late follicular and midluteal phases [28]. Despite a 3.5% increase in fluid retention during the midluteal phase, the variance in menstrual hormones did not have a significant impact on electrolyte restoration [28]. 

The study measuring athletic outcomes only in the luteal phase reported the impact of water ingestion on thermoregulation and soccer-specific skills such as passing a soccer ball while maneuvering around a grid of cones as quickly as possible (as measured by the Loughborough Soccer Passing Test) [38]. Fluid ingestion reduced the participants’ perceived exertion to 86% of the exertion experienced in the non-hydrated trial, and mean blood lactate levels were 51% lower in the soccer trials receiving fluid [38]. Despite the favorable physiological outcomes, the fluid intervention only improved the performance time for the Loughborough Soccer Passing Test by <5 s, which was not statistically significant [38].

Of the nine studies that measured outcomes only in the follicular phase, two measured the impact of a carbohydrate mouth rinse [31,32]. The first study reported that a 5 s carbohydrate mouth rinse did not significantly improve endurance capacity in female recreational runners; the distance traveled in a 60 min run after the mouth rinse only increased by 1.6% [31]. The second study, which utilized non-endurance-trained female participants, reported that a 5 s carbohydrate mouth rinse reduced participants’ perceived exertion by nearly 12% in a 65 min exercise bout performed at 75% maximal oxygen uptake (VO_2peak_) and significantly attenuated post-exercise executive functional decline by improving reaction time by 9 ms, as measured by a modified incongruent Stroop Color and Word Test [32]. 

Five of the rehydration therapies that were used for intervention during the follicular phase utilized carbohydrate–electrolyte solutions composed of 3–6% carbohydrate with sodium and potassium [29,33,34,35,36], and three of those compared these solutions with other types of water [29,35,36]. In female participants with moderate to excellent aerobic capacity, deep ocean mineral water restored unstimulated saliva osmolality nearly 30 min faster than the commercial carbohydrate-based beverage and spring water [29]. In both amateur cyclists and recreationally active women, plain water improved markers of dehydration (loss of body mass, body temperature, urine volume, urine specific gravity) to a similar extent as salt water, as well as 3% or 6% carbohydrate-containing beverage (fluid retention was 68%, 72%, 68%, and 76%, respectively) [35,36]. In recreationally active participants [34] and experienced runners [33], a carbohydrate–electrolyte solution improved running times by 16% and 3.7%, respectively. Interestingly, the ergogenic effect of this solution was reduced by the addition of protein by nearly 3 min, and the carbohydrate–electrolyte protein solution yielded time trial results statistically similar to the placebo results [33]. 

The two remaining studies measuring outcomes in the follicular phase used electrolyte interventions for physically active participants. Participants received pickle juice and mustard to determine the impact of sodium concentration on dehydration markers; however, neither pickle juice nor mustard improved plasma osmolality or plasma volume significantly after dehydration [30]. Between sodium phosphate and the placebo intervention, only a 1.3% difference in VO_2peak_ was measured, which was not statistically different [37]. 

Despite intra-menstrual cycle variability not directly influencing markers of dehydration or net fluid balance [28], certain rehydration therapies did improve athletic performance. These therapies included deep ocean mineral water [29] and a 6% carbohydrate–electrolyte solution (CES) [33,34]. Although CES rehydration improved endurance capacity by increasing time to exhaustion [34] and reducing overall time trial performance [33], standalone electrolyte interventions did not significantly alter fluid restoration [39] or improve anaerobic or aerobic thresholds [37]. Although rehydration therapies attenuated physiological markers of exercise-induced dehydration [35,36,37,38], results varied between enhancing athletic performance [29,32,33,34] and having no significant impact [31,35,37,38].

### 3.2. Micronutrient Interventions 

Among the studies involving micronutrient interventions (Table 2), one study was conducted in Japan [39] and one in the United States [40]. Both studies included endurance-trained athletes [39,40]. Due to the length of intervention time, one study controlled for menstruation by having women record their menstrual status daily [40]. The second study performed trials during specific menstrual phases to reduce experimental variability [39]. 

One study evaluated the outcomes of multi-week iron supplementation on iron-depleted, nonanemic (IDNA) participants [40]. In endurance-trained IDNA female rowers, iron supplementation, in addition to training, improved energy efficiency (measured via indirect calorimetry) during a 4 km time trial by 1.3% [40]. The energy expenditure difference between the IDNA supplementation group and IDNA placebo group for the 4 km time trial was 15 kilocalories over the 20 min [40]. From the daily recording of menstrual status throughout the supplementation period, menstrual status was not reported to affect energy expenditure in the iron supplementation or placebo groups [40]. 

The second study evaluated a calcium-based intervention on trained female cyclists to determine the effect on time trial performance and exercise-induced bone resorption [39]. A pre-exercise calcium-rich meal did not improve or impair a 10 min time trial, maintaining the highest power possible after 80 min of cycling [39]. The calcium-rich meal did, however, reduce biochemical markers of bone resorption (cross-linked c-telopeptide of type I collagen) by approximately 25% when compared with the control group [39]. The intervention and control groups were balanced in regard to the menstrual status of participants in their luteal or follicular phase [39]. 

Of the micronutrient-based interventions, one of the studies reported an increase in athletic performance directly [40], and the other by modulating exercise-induced damage by reducing bone resorption biomarkers [39]. Iron supplementation improved energy efficiency and expenditure [40]. Although calcium did not directly affect time trial performance, the intervention attenuated bone resorption [39], having positive implications for females participating in endurance activities through the modulation of exercise-induced damage. Based on these reported findings, iron and calcium supplementation seem to improve or support the performance of endurance female athletes through the menstrual cycle. 

### 3.3. Omega-3-Fatty Acids and Phytochemical-Based Dietary Supplement Interventions 

As outlined in Table 3, one study assessed omega-3-fatty acids [41], and nine studies assessed phytochemical-based dietary supplement interventions [42,43,44,45,46,47,48,49,50]. Three of these studies originated from the United Kingdom [42,45,47], three from Spain [43,44,46], three from Australia [48,49,50], and one from the United States [41]. Three studies measured outcomes during the follicular phase [47,48,49], three studies measured in the luteal phase [42,45,46], and three studies took measurements in multiple menstrual phases and compared the results from each phase [41,43,44]. The tenth study recorded the menstrual cycle over a three week period and recorded testing either in the mid-luteal phase or “other” [50]. 

Four interventions utilized caffeine and included competitive athletes [44], endurance-trained participants [43], physically active participants [46], and amateur team sport athletes [48]. Strength-based outcomes were assessed in competitive athletes who consumed caffeine during their early follicular phase, late follicular phase, and mid-luteal phase [44]. Caffeine increased the mean velocity of a half-squat at 60% of one-repetition maximum (1-RM) in both the early follicular phase and late follicular phase by 1.4 ± 2.7% and 5.0 ± 10.4%, respectively [44]. In the mid-luteal phase, the reported ergogenic effect of caffeine was non-significant [44]. Anaerobic power was assessed in triathletes who consumed caffeine during their early follicular phase, preovulatory phase, and midluteal phase [43]. Across all three phases, caffeine increased anaerobic cycling power performance by nearly 2% in peak and mean power generated [43]. In physically active participants tested only in their luteal phase, caffeine increased maximal fat oxidation by nearly 32% and reduced perceived exertion by nearly 18% compared to the placebo, and nearly 27% compared to *p*-synephrine during an incremental intensity exercise test [46]. *p*-Synephrine is a naturally occurring alkaloid found in bitter oranges and has been shown to increase fat oxidation during submaximal exercise. Despite this metabolic effect, *p*-synephrine lacked a strong independent effect on fat oxidation when combined with caffeine [46]. In amateur team sport athletes, caffeine ingestion alone had no significant effect on repeated 20 m sprint performance. However, caffeine ingested with sodium phosphate improved the best 20 m sprint performance in the third set by 6% when compared with the placebo trial [48].

Four interventions measured the impact of anthocyanins; three of which utilized New Zealand blackcurrant supplementation [42,47,50], and the fourth using Montmorency tart cherry supplementation [45]. Blackcurrants were reported to increase fat oxidation, determined through expired air collected into Douglas bags, during submaximal exercise in recreationally active participants by 18% without a change in body temperature [42]. In endurance-trained athletes, blackcurrant supplementation similarly increased fat oxidation by 27% during submaximal exercise [47]. Blackcurrant juice had less of an impact on running times, but was shown to improve 5 km time trial performance in runners with a faster baseline running performance [50]. Slower runners with a lower training load, on the other hand, did not experience the same ergogenic effect of blackcurrant juice during the 5 km time trial [50]. With respect to the menstrual phase, results in this study were reported as taking place in the mid-luteal phase (day 18–24, inclusive of menses) or “other.” In both the time trial performance and laboratory performance, the menstrual phase had a minor influence on results, but the significance was not reported [50]. Montmorency tart cherry supplementation in university dancers did not impact 30 m sprint times; however, the intervention improved the pain pressure threshold by accelerating the recovery of muscle function as measured by a 100% return to a countermovement jump performance within 72 h post-exercise [45].

One study assessed beetroot juice supplementation in the follicular phase [49]. In amateur team sport athletes, 70 mL of beetroot juice did not significantly improve sprint ability [49]. 

The remaining study intervened with fish oil supplementation and tested during two menstrual phases to compare results [41]. In response to an eccentric exercise protocol, physically active women did not experience a significant difference in perceived muscle soreness between the mid-follicular and mid-luteal phase; however, fish oil increased perceived soreness by approximately 38% when compared to the placebo group in both phases [41]. 

Caffeine as an ergogenic aid increased anaerobic leg power in three menstrual phases [43], increased mean peak strength in two menstrual phases [44], and increased fat oxidation in the follicular phase [46]. Blackcurrant extract enhanced submaximal substrate utilization in both the mid-luteal phase [42] and follicular phase [47]; however, the supplementation did not affect oxygen uptake in either of the phases. Beetroot juice had little impact on sprint ability when tested in the follicular phase [49]. Menstrual phase variability did not impact perceived muscle soreness, whereas fish oil supplementation increased soreness across phases [41]. Based on these reported findings, the menstrual phase seems to influence the efficacy of phytochemical-based dietary supplementation, and future research should compare athletic outcomes across the menstrual cycle. 

## 4. Discussion 

The menstrual cycle is an important indicator for women’s health and physiology, reflecting hormone balance and nutritional status. As a component of women’s health, menstruation affects performance in women who are untrained, recreationally active, and elite professionals. With regard to exercise, menstruation has been shown to influence aerobic performance in trained [4] and untrained women [3]. Trained women experience reduced aerobic capacity (measured as VO_2peak_) during their luteal phase [4], and untrained women experience a reduced cycling power output during their mid-luteal phase [3]. Anaerobic exercise tests, on the other hand, including start speed or power performance, have not been shown to be influenced by the menstrual phase [51]. Key findings from this review highlight the efficacy of certain nutrition-based interventions in specific menstrual phases. Findings from this review also offer new perspectives on when during the menstrual phase nutrition-based interventions may be most effective. These new perspectives are limited, however, because many of the athletic performance findings were reported in only one menstrual phase. 

### 4.1. Hydration Interventions 

Among the studies examining hydration interventions, we identified the role of carbohydrate–electrolyte solutions (CESs) in improving time-trial performance [33] and increasing exercise time to exhaustion [34] in the follicular phase. Of note, the effectiveness of the CES interventions was only tested in the follicular phase for both studies. The CES beverages included 6% carbohydrate content, totaling 24 calories per 100 mL [33,34]. Glucose is known to delay gastric emptying due to its caloric content and osmolality [52]. Electrolytes similarly contribute to the osmotic gradient between plasma and the large intestine, increasing net water movement into the intestine [53]. By reducing the rate of water loss while also changing the osmotic concentrations, and promoting water absorption, CES therapies mechanistically promote euhydration throughout bouts of exercise. Fluid retention during exercise supports aerobic performance by preventing a decline in stroke volume and cardiac output, ultimately mitigating cardiovascular drift [54]. 

The application of CES to improve aerobic performance in the luteal phase is less clear. Studies measuring gastric emptying rates along with sex hormone concentration have reported inconsistent findings between the luteal and follicular phases [55]. The influence of sex hormones on gut permeability has not been extensively studied. However, progesterone has been reported to decrease gut permeability by increasing trans-epithelial electrical resistance [56]. With a reduction in large intestine mucosa permeability, the luteal phase may hinder the success of CES rehydration therapies. 

Sex hormone variability does not seem to influence fluid and electrolyte restoration after dehydration [28], or absolute stroke volume during a graded exercise test [57]. Despite a comparable absolute stroke volume between the luteal and follicular phase, stroke volume had a larger negative relative change in the luteal phase [57]. Considering the reduction in aerobic performance in the luteal phase [3,4] and the influence of progesterone on intestinal permeability [56], intra-exercise rehydration should further be evaluated in the luteal phase. Interventions accounting for differences in luteal phase gastric emptying or gut permeability may mitigate the greater decrement in stroke volume percent change when compared to the follicular phase. 

### 4.2. Micronutrient Interventions

Of the studies assessing micronutrient interventions, we identified the role of iron in improving endurance [40]. In the aerobic trial, non-anemic women received 30 mg of elemental iron [40]. Iron recommendations for treating iron deficiency (ID) and iron deficiency anemia (IDA) range between 80 and 200 mg elemental iron [58]. The recommended iron dosage for premenopausal women is 18 mg per day [59]. Despite supplementing with a dosage below the recommended amount for treating iron deficiency, the study still reported an improvement in iron stores with the 30 mg supplementation [40]. 

In animal models, dietary iron deficiency reduces the ability of skeletal muscle tissue to consume oxygen and produce adenosine triphosphate (ATP) due to decreased mitochondrial dehydrogenase activity [60]. The reduction in mitochondrial enzymatic activity limited endurance capacity in iron-depleted rats; however, with iron repletion, endurance capacity improved after five days of supplementation [60]. In the same study, oxygen uptake improved only three days after iron repletion, likely due to the rapid recovery in hematocrit and hemoglobin levels [60]. Iron status is closely correlated to skeletal muscle mitochondrial function and oxygen-carrying capacity, thus influencing aerobic athletic performance. 

Regular menstrual blood loss has not been reported to influence hemoglobin mass; however, oral contraceptives increase serum iron levels, potentially contributing to increased oxygen-carrying capacity [61]. Given the relationship between oral contraceptives and serum iron levels, the need for or dosage of iron supplementation may vary for female athletes, depending on their contraceptive use. Future studies should evaluate what amount of iron supplementation is necessary to promote a comparable oxygen-carrying capacity for women not taking oral contraceptives. 

We also identified the role of calcium in favorably modulating exercise-induced bone resorption by reducing parathyroid hormone (PTH) immediately after exercise and 40 min after exercise [39]. The intervention was provided as a single pre-exercise calcium-rich meal with approximately 1352 mg of calcium, which is 135% of the daily recommended intake for premenopausal women [62]. In mice, a single bout of exercise can increase circulating PTH [63], which has been reported to stimulate a reduction in osteoblasts and proliferation of osteoclasts in cellular models [64]. PTH secretion is also stimulated by a reduction in serum calcium concentrations. In animal models, induced hypocalcemia increased PTH levels 10- to 12-fold to restore serum calcium homeostasis [65]. Previous studies suggest that bone resorption (as measured by serum deoxypyridinoline) increases in the follicular phase due to reduced progesterone and relative estrogen concentration [66]. The calcium-based meal, however, was given to groups balanced between follicular and luteal phases, suggesting a positive influence over bone resorption, independent of sex hormone variation [39]. 

### 4.3. Omega-3-Fatty Acids and Phytochemical-Based Dietary Supplement Interventions

Among the studies assessing phytochemical-based dietary supplement interventions, we identified the role of 3 mg of caffeine per kg body weight in improving anaerobic power generation [43], mean peak strength velocity [44], and the perception of muscle power and endurance during exercise [46]. Muscular contraction relies on extracellular calcium concentration, and in skeletal muscle, calcium ions enter the cytosol from the sarcoplasmic reticulum [67]. Caffeine has been shown to increase the release of calcium ions from the sarcoplasmic reticulum of frog muscles [68] while also increasing muscular contraction force through increased calcium sensitivity in mice [69]. Caffeine intake has been suggested to impact plasma concentrations of female sex hormones and increase testosterone levels [70]. However, due to methodological inconsistencies, conflicting results exist in the literature on the impact of female sex hormones and skeletal muscle contraction. The power and velocity improvements measured throughout the menstrual cycle, however, suggest that caffeine enhances anaerobic performance regardless of female sex hormone concentration. 

Anthocyanin-rich supplementation had differential effects on performance outcomes, whereby blackcurrants had little influence on oxygen uptake despite increased fat oxidation [42,47], and beetroot juice had little impact on sprint performance [49]. Despite reporting similar results, the two trials with blackcurrant supplementation provided differing doses of anthocyanin: 67 mg [47] and 105 mg [42], suggesting a plateau in the dose–response relationship of blackcurrant supplementation.

In animal models, anthocyanins have been reported to up-regulate lipid metabolism by enhancing the expression of fatty acid synthase, glycerol-3-phosphate acyltransferase, 3-phosphate dehydrogenase, hormone sensitive lipase, and perilipin gene expression [71]. This interaction between adipocytes and anthocyanins may explain the increases in fat oxidation during exercise when supplemented with blackcurrants [42,47]. Future studies should evaluate time to exhaustion in females to assess the impact of increased fat oxidation on submaximal athletic performance. In animal models, organic nitrates induced vasodilation by stimulating cyclic guanosine monophosphate (cGMP) accumulation and relaxing vascular smooth muscle [72]. Clinical applications of vasodilators have reported increases in cardiac output due to reduced peripheral resistance [73], and the menstrual cycle has been reported to impact flow mediated vasodilation [74]. High estradiol levels increase flow-mediated diameter [74]; therefore, the impact of high-dose dietary nitrate supplementation should also be assessed during the follicular phase to determine any auxiliary effect.

Fish oil supplementation increased soreness across menstrual phases [41]. The supplementation provided 2.4 g of eicosapentaenoic acid (EPA) and 1.8 g of docosahexaenoic acid (DHA), which are 960% and 720% of daily recommended values (0.25 g each), respectively [75]. Studies assessing fish oil have reported inconsistent results concerning muscle soreness [41], and some data suggest that despite the modulation of exercise-induced damage by fish oil, soreness is not reduced [76]. Estrogen has an inverse relationship with creatine kinase, a marker of mechanical muscle damage, suggesting protection against muscle soreness [77]. However, our findings do not support this relationship, because women supplementing fish oil still reported a high perception of soreness, despite increased serum estradiol levels [41]. Reduced oxidative stress may not directly correlate to reduced soreness [76]; therefore, the role of fish oil in modulating muscle soreness for female athletes throughout the menstrual cycle needs future attention. 

### 4.4. Strengths and Limitations 

This paper has several strengths. First, the authors addressed the lack of a focused review on the implications of menstruation on nutrition-based interventions influencing athletic performance outcomes or modulating exercise-induced damage by identifying and evaluating randomized controlled trials and crossover studies. Secondly, the authors conducted a structured literature search and focused selection process using stringent eligibility criteria. Thirdly, 97% of the included manuscripts (22) were randomized trials and 83% (19) included at least one control or placebo group. Finally, a variety of interventions were recorded in the trials, including rehydration therapies, as well as micronutrient (calcium, iron), omega-3-fatty acids, and phytochemical-based dietary supplementation.

The present review also has a few limitations. Firstly, our review is a critical focused review that was not intended to be a qualitative systematic review or quantitative meta-analysis. We did not perform statistical analysis on data from separate studies due to the variation in outcomes measured and interventions used. The purpose of this review is to provide readers an overview of multiple relevant nutrition-based interventions and report the relevant findings. Secondly, the literature search only used PubMed, Medline, and Google Scholar; however, these platforms are considered major repositories of published health studies. Thirdly, the only studies evaluated were written in English. Additionally, the one study intervening with iron supplementation did not report the specific menstrual phase in which testing took place, and instead recorded the participants’ cycle information during the supplementation time frame. This study was still included because the menstrual cycle was reported to not impact outcomes in the intervention or control group. This methodological shortcoming, however, does not address the underlying question of how menstrual phase influences performance and supplementation, highlighting the need for future research relating menstrual phase to supplementation outcomes. Finally, although all included studies reported a specific menstrual phase for testing or recorded menstrual cycles for participants, only five studies assessed outcomes in more than one phase. This limitation emphasizes the need for additional inquiries into the effects of the variance in female sex hormones on nutrition interventions influencing athletic outcomes. 

### 4.5. Future Directions 

The outcomes of anaerobic muscular power, aerobic cardiovascular fitness, and modulation of exercise-induced dehydration and muscle damage evaluated in the present review are conventional measurements of exercise performance. The historic omission of female participation in exercise research [8], however, has rendered the impact of female physiology on these measurements opaque. Keeping this in mind, we acknowledge that the results of this review inform readers of the interventions that have been shown to help eumenorrheic female athletes during a menstrual phase but do not outline the mechanistic relationship with female sex hormones or consider dysmenorrhea. Therefore, we recommend that future investigations include women in interdisciplinary exercise and nutrition science original research, comparing results across the menstrual phases. Future focus on the interaction between menstrual cycle variance and nutrition interventions will better support women participating in sport recreationally and professionally. 

## 5. Conclusions

**Figure 2:**
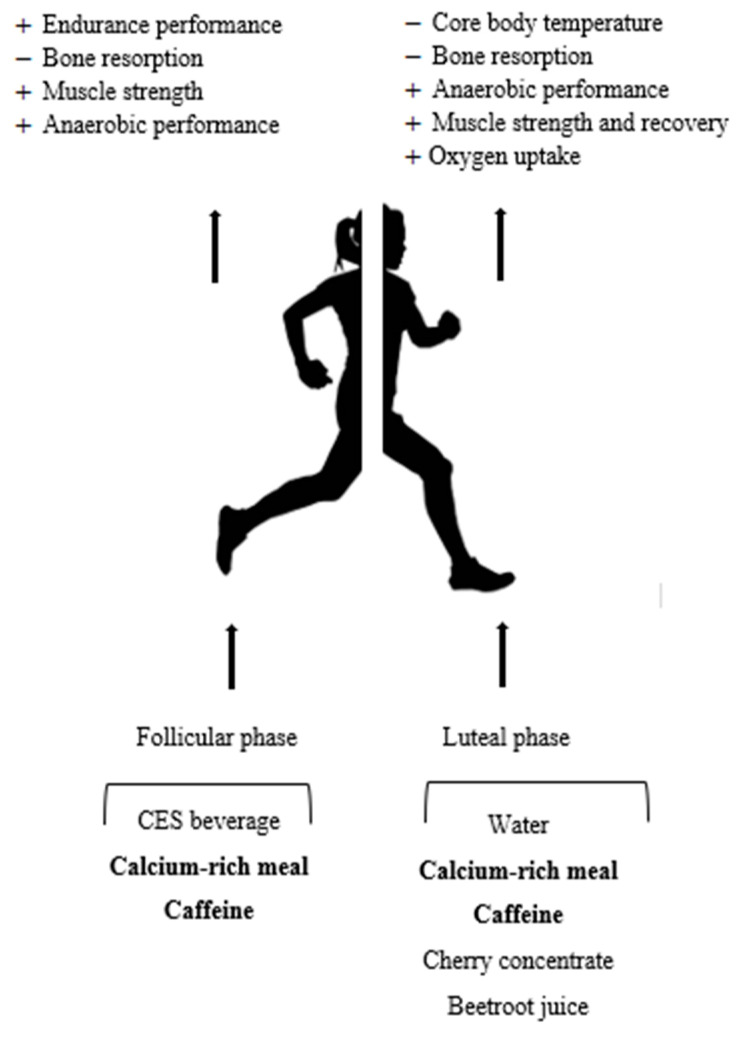
Effects of carbohydrate-based rehydration, as well as micronutrient and phytochemical-based supplementation on athletic performance through the menstrual phase. + Indicates enhancement; − Indicates reduction; CHO: carbohydrate; CES: carbohydrate–electrolyte solution; bolded interventions tested in multiple menstrual phases during same trial.

The present review is a focused critical analysis of 23 studies that aims to inform readers on the association between nutrition-based interventions and athletic performance in female participants, with relation to the menstrual cycle. Although many trials only addressed one menstrual phase, promising interventions for the follicular phase included: (1) CES beverages for enhanced endurance performance; and (2) blackcurrant extract for enhanced fat oxidation. Promising interventions only tested in the luteal phase include: (1) Montmorency cherry concentrate for improvements in the pain pressure threshold; and (2) caffeine for enhanced fat oxidation. The application of these findings is inherently limited due to athletic testing only completed in one menstrual phase. Due to the hormonal fluctuation throughout the menstrual cycle, a nutrition-based intervention in one phase may not produce the same outcomes in the rest of the cycle. Of the few trials measuring outcomes in multiple menstrual phases for comparison across the cycle, promising interventions include: (1) pre-exercise calcium-rich meals on modulating exercise-induced bone resorption; and (2) caffeine on peak power output and muscle strength. By understanding the physiology of menstruation for a female athlete, cycle-specific nutrition-based interventions can improve athletic performance (Figure 2). Although intra-cycle variability does not seem to influence dehydration levels, muscular strength, or perceived muscle soreness, many advantageous interventions have only been tested in one menstrual phase. With the growing participation of women in sport, the need to better understand interventions to mitigate the impacts of menstruation on exercise performance is an urgent area of research. Similarly, a better understanding of when during the menstrual phase interventions will be most successful should be further explored. Profound improvements in female athletic performance are likely possible with future effective nutrition-related investigations.

## Figures and Tables

**Table 1:** Studies reporting on the effects of hydration-based interventions on athletic performance in menstruating women.

Authors, Year (Country)	Study Design	Participants	Menstrual Cycle Reported	Nutrition-Based Intervention and Duration	Assessment of Athletic Outcome
Rodriguez-Giustiniani and Galloway, 2019 (United Kingdom) [28]	Crossover study	Women (*n* = 10) Age: 25 ± 7 years	LFP: between days 10 and 13 MLP: between days 18 and 23	100% body mass loss volume: 6.4% CHO, 25 mM Na^+^, 3.5 mM K^+^ beverage Consumed in four equal phases over 30 min	↑ Fluid retention in LFP (trivial effect) ∅ Menstrual phase on degree of dehydration, urine volume, net fluid balance, electrolyte balance, urine osmolality, thirst intensity
Harris et al., 2019 (United States) [29]	Randomized counterbalanced crossover study	Women (*n* = 8) Age: 21 ± 2 years	Early in cycle	100% body mass loss volume: Deep-ocean mineral water 59.2 g/L CHO, 450.9 mg/L Na^+^, 408.3 mg/L Cl^−^, 126.8 mg/L K^+^ beverage Spring water Consumed in two phases 30 min apart	Rehydrating with deep-ocean mineral water: ↑ Peak torque ↓ Salivary osmolality
Chryssanthopoulos et al., 2018 (Greece) [31]	Double-blind placebo-controlled RCT	Women (*n* = 15) Age: 43 ± 2 years	FP: between days 3 and 10	25 mL 6.4% CHO beverage Rinsed in mouth for 5 s prior to exercise and at minute 15, 30, and 45	∅ Distance traveled, HR, fluid loss
Konishi et al., 2017 (Japan) [32]	Single-blind RCT	Women (*n* = 4) Age: 24 ± 2 years	FP	25 mL 6.4% maltodextrin solution Rinsed in mouth for 5 s prior to exercise	↓ Reaction time, RPE plasma E and NE ∅ Executive function accuracy, plasma ACTH
Gui et al., 2017 (Hong Kong) [33]	Randomized, placebo-controlled crossover study	Women (*n* = 11) Age: 32 ± 7 years	Within 10 days after menses ended	150 mL 6% CES or 150 mL 4% CHO + 2% PRO CES-P Consumed every 2.5 km for 21 km run	CES: ↓ 21 km time CES-P: ∅ 21 km time CES and CES-P: ∅ USG, RPE, cognitive reaction time
Sun et al., 2015 (China) [34]	Double-blind placebo-controlled RCT	Women (*n* = 8) Age: 28 ± 2 years	FP	3 mL·kg^−1^ body mass 6% CES Consumed every 20 min until exhaustion	↑ Exercise time to exhaustion, plasma glucose from 15 min mark ∅ RER, blood glucose, lactate levels, HR, RPE, PTS, PAS
Miller, 2014 (United States) [30]	Randomized, crossover study	Women (*n* = 6) Age: 25 ± 2 years	FP	1 mL·kg^−1^ body mass pickle juice Bolus of mustard with similar [Na^+^] to pickle juice Consumed in full in 2.5 min	∅ Plasma Na^+^ or K^+^ concentration, plasma osmolality, plasma volume
Ramos-Jiménez et al., 2014 (Mexico) [35]	RCT	Women (*n* = 9) Age: 24 ± 5 years	FP	100% of body mass loss: Plain water hydration or 324 mmol/L CHO, 19.9 mmol/L Na^+^, 3.2 mmol/L K^+^ beverage Consumed every 15 min for 90 min	Both water and CHO-based beverage: ↓ Loss of body mass, body temperature, mean blood pressure, HR ∅ Distance traveled, resistance applied to ergometer
Logan-Sprenger and Spriet, 2013 (Canada) [36]	Randomized, crossover study	Women (*n* = 6) Age: 25 ± 1 years	FP	600 mL of each: Water 40 mM Na^+^ salt water 3% CES 6% CES Consumed in two phases 15 min apart	Starting in a hypohydrated state, all 4 beverages: ↓ USG ↓ Urine volume
West et al., 2012 (Australia) [37]	Double-blind placebo-controlled counterbalanced RCT	Women (*n* = 9) Age: 23 ± 3 years	FP: between days 1 and 5	50 mL·kg^−1^ fat-free mass of sodium phosphate Consumed daily for 6 days with fluid	∅ VO_2peak_, running speed, HR
Ali et al., 2011 (New Zealand) [38]	Randomized, crossover study	Women (*n* = 10) Age: 26 ± 5	LP	3 mL·kg^−1^ body mass water Consumed every 15 min for 90 min	↓ Change in body mass, core body temperature, HR, blood lactate concentration, RPE ∅ Sprint performance

Crossed circle (∅) indicates no effect. Up arrow (↑) indicates an increase and down arrow (↓) indicates a decrease. ACTH: adrenocorticotropic; CHO: carbohydrate; CES: carbohydrate–electrolyte solution; CES-P: carbohydrate–electrolyte protein solution; Cl^−^: chloride; E: epinephrine; FP: follicular phase; g: gram; HR: heart rate; K^+^: potassium; kg: kilogram; km: kilometer; L: liter; LFP: late follicular phase; LP: luteal phase; mg: milligram; min: minute; mL: milliliters; MLP: midluteal phase; mM: millimolar; Na^+^: sodium; NE: norepinephrine; PAS: perceived abdominal discomfort scale; PRO: protein; PTS: perceived thirst scale; RER: respiratory exchange ratio; RCT: randomized controlled trial; RPE: rating of perceived exertion; s: second; USG: urine specific gravity; VO_2peak_: maximal oxygen uptake.

**Table 2:** Studies reporting on the effects of micronutrient-based interventions on athletic performance in menstruating women.

Authors, Year (Country)	Study Design	Participants	Menstrual Cycle Reported	Nutrition-Based Intervention and Duration	Assessment of Athletic Outcome
Haakonssen et al., 2015 (Japan) [39]	Randomized counterbalanced crossover study	Women (*n* = 32) Age: 24 ± 4 years	LP or FP	Pre-exercise meal with 1352 ± 53 mg calcium Consumed 2 h before exercise	↓ Exercise-induced bone resorption markers, hematocrit percentage ∅ Sweat calcium levels, 10 min time trial
Dellavalle and Haas, 2013 (United States) [40]	Double-blind placebo-controlled RCT	Women (*n* = 31) Age: 20 ± 1 years	Menstrual status quantified daily	50 mg iron sulfate Consumed twice per day for 6 weeks	↑ Gross efficiency, absolute VO_2peak_, maximal work rate ↓ Energy expenditure, maximal blood lactate concentration ∅ Endurance time trial, relative VO_2peak_, HR maximum, RER

Crossed circle (∅) indicates no effect. Up arrow (↑) indicates an increase and down arrow (↓) indicates a decrease; FP: follicular phase; h: hour; HR: heart rate; LP: luteal phase; mg: milligram; min: minute; RCT: randomized controlled trial; RER: respiratory exchange ratio; VO_2peak_: maximal oxygen uptake.

**Table 3:** Studies reporting on the effects of omega-3-fatty acids and phytochemical-based dietary supplements on athletic performance in menstruating women.

Authors, Year (Country)	Study Design	Participants	Menstrual Cycle Reported	Nutrition-Based Intervention and Duration	Assessment of Athletic Outcome
Hiles et al., 2020 (United Kingdom) [42]	Randomized, placebo-controlled double-blind crossover study	Women (*n* = 6) Age: 21 ± 2 years	MLP	300 mg New Zealand BC extract Consumed twice daily for 7 days	↑ Fat oxidation ↓ RER, CHO oxidation ∅ HR, VO_2_, VCO_2_; rectal, skin, body temperature; whole body sweat rate
Lara et al., 2020 (Spain) [43]	Double-blind, placebo-controlled, crossover RCT	Women (*n* = 13) Age: 31 ± 6 years	EFP, preovulatory phase, MLP	3 mg·kg^−1^ body mass caffeine Consumed 60 min prior to exercise	In EFP, preovulatory phase, MLP: ↑ 15 s Wingate peak power
Romero-Moraleda et al., 2019 (Spain) [44]	Double-blind placebo-controlled crossover RCT	Women (*n* = 13) Age: 31 ± 6 years	EFP LFP MLP	3 mg·kg^−1^ body mass caffeine Consumed 45 min prior to exercise	In EFP and LFP: ↑ Peak velocity at 60% 1-RM
Brown et al., 2019 (United Kingdom) [45]	Double-blind placebo-controlled RCT	Women (*n* = 20) Age: 19 ± 1 years	ELP to MLP or 14 days before withdrawal bleed	30 mL Montmorency cherry concentrate Consumed twice daily for 8 days	↑ Pain pressure threshold at rectus femoris, CMJ muscle recovery ↓ Rating of muscle soreness ∅ Hamstring stiffness and flexibility, maximum voluntary isometric contraction, 30 m sprint time, repeated sprint time, RPE
McKinley-Barnard et al., 2018 (United States) [41]	Double-blind placebo-controlled RCT	Women (*n* = 22) Age: 21 ± 1 years	MFP: day 6 MLP: day 21	2.4 g EPA and 1.8 g DHA (FO) Consumed daily for 21 days	FO: ↑ Perceived muscle soreness, serum estradiol FO during MFP: ↓ Serum myoglobin FO and cycle phase: ∅ Muscular strength Cycle phase: ∅ Perceived muscle soreness
Gutierrez-Hellin and Del Coso, 2018 (Spain) [46]	Double-blind placebo-controlled RCT	Women (*n* = 2) Age: 25 ± 7 years	LP	3 mg·kg^−1^ caffeine 3 mg·kg^−1^ *p*-synephrine Consumed 60 min prior to exercise	Caffeine: ↑ Fat oxidation at 30–70% VO_2max_ Caffeine + *p*-synephrine: ↑ Fat oxidation at 40% and 70% VO_2max_ Caffeine: ↑ Muscle power and endurance perception Caffeine: ↓ CHO oxidation at 70% VO_2max_ Caffeine: ↓ Perceived exertion *p*-synephrine: ↓ CHO oxidation at 60% VO_2max_ ∅ Energy expenditure
Strauss et al., 2018 (United Kingdom) [47]	Randomized, placebo-controlled double-blind crossover study	Women (*n* = 16) Age: 28 ± 8 years	FP: between days 9 and 11	600 mg·day^−1^ New Zealand BC extract Consumed daily for 7 days	↑ Fat oxidation ↓ CHO oxidation ∅ HR, VO_2_, VCO_2_
Buck et al., June 2015 (Australia) [49]	Randomized, placebo-controlled double-blind Latin-square design	Women (*n* = 13) Age: 26 ± 2 years	FP	50 mg·L^−1^ SP Consumed daily for 6 days 70 mL concentrated BJ Consumed 3 h prior to exercise	SP: ↓ Set 1, 2, overall total sprint time, best sprint time SP + BJ: ↓ Set 2 total sprint time vs. placebo BJ: ∅ total sprint time, best sprint time ∅ HR, RPE, blood lactate
Buck et al., March 2015 (Australia) [48]	Randomized, placebo-controlled double-blind Latin-square design	Women (*n* = 12) Age: 26 ± 2 years	FP	50 mg·L^−1^ SP Consumed daily for 6 days 6 mg·kg^−1^ body mass caffeine Consumed 1 h prior to exercise	SP + Caffeine: ↓ Set 1, 2, 3, and overall total sprint time vs. placebo SP + Caffeine: ↓ Set 3 and overall total sprint time vs. Caffeine and vs. SP SP: ↓ Set 1 and 3 total sprint time vs. placebo SP + Caffeine: ↓ Best sprint time ∅ HR, RPE
Braakhuis et al., 2014 (Australia) [50]	Randomized, placebo-controlled crossover study	Women (*n* = 23) Age: 31 ± 8 years	Cycle recorded over 3 weeks	0.5 L VC juice or BC juice Consumed daily for 21 days	VC: ↓ Training speed VC and BC: ↑ Running times BC: ↓ 5 km time trial in fast runners

Crossed circle (∅) indicates no effect. Up arrow (↑) indicates an increase and down arrow (↓) indicates a decrease. BC: blackcurrant; BJ: beetroot juice; CHO: carbohydrate; CMJ: countermovement jump; DHA: docosahexaenoic acid; EFP: early follicular phase; ELP: early luteal phase; EPA: eicosapentaenoic acid; FO: fish oil; FP: follicular phase; h: hour; HR: heart rate; kg: kilogram; km: kilometer; L: liter; LFP: late follicular phase; LP: luteal phase; m: meter; MFP: mid-follicular phase; mg: milligram; min: minute; mL: milliliter; MLP: mid-luteal phase; 1-RM: one repetition maximum; RCT: randomized controlled trial; RER: respiratory exchange ratio; RPE: rating of perceived exertion; s: second; SP: trisodium phosphate dodecahydrate; VC: vitamin C; VCO_2_: carbon dioxide production; VO_2_: oxygen uptake; VO_2max_: maximal oxygen uptake.
